# Adenomyoma recurrence 7 years after laparoscopic supracervical hysterectomy: A case report and literature review

**DOI:** 10.1097/MD.0000000000036089

**Published:** 2023-11-17

**Authors:** Chin-Tzu Tien, Dah-Ching Ding

**Affiliations:** a Department of Obstetrics and Gynecology, Hualien Tzu Chi Hospital, Buddhist Tzu Chi Medical Foundation, and Tzu Chi University, Hualien, Taiwan; b Institute of Medical Sciences, Collagen of Medicine, Tzu Chi University, Hualien, Taiwan.

**Keywords:** adenomyoma, dysmenorrhea, myoma, recurrence, supracervical hysterectomy

## Abstract

**Rationale:**

Adenomyosis, a gynecological condition characterized by endometrial tissue within the uterine myometrium, often leads to menstrual pain and heavy bleeding, significantly affecting the quality of life. The primary treatment for adenomyosis and leiomyomas is hysterectomy. However, in rare instances, these conditions can recur in the cervical stump following a hysterectomy.

Here, we present a case of cervical adenomyoma development after a prior laparoscopic supracervical hysterectomy.

**Patient Concerns:**

A 47-year-old woman sought medical attention due to increased vaginal bleeding.

**Diagnoses:**

She had undergone a laparoscopic supracervical hysterectomy 7 years earlier to address uterine myoma and adenomyosis. Just 1 month posthysterectomy, a pelvic ultrasound revealed the presence of a cervical stump measuring approximately 4.0 × 4.0 cm. Subsequent follow-up ultrasounds documented the gradual growth of the cervical mass. Two years ago, a recurrent myoma was identified, and the patient experienced intermittent vaginal bleeding. Over 7 years, the cervical mass increased from 4 to 7 cm. Preadmission pelvic ultrasonography confirmed the existence of cervical adenomyoma measuring 7 × 6 cm.

**Interventions:**

Consequently, the patient underwent a laparoscopic trachelectomy. Intraoperatively, an enlarged cervix, approximately 7 × 6 cm in size, containing adenomyoma was observed. A gross examination of the specimen indicated hypertrophic muscle tissue and hemorrhagic foci. Subsequent histopathological examination confirmed the presence of adenomyoma.

**Outcomes:**

Remarkably, the patient exhibited no recurrence over the subsequent 8 months.

**Lessons:**

The case presented here highlights the potential occurrence of cervical adenomyoma following a supracervical hysterectomy. Management options include hormone therapy and surgical excision. Furthermore, annual follow-up comprising ultrasound and pap smear evaluations is recommended for patients with supracervical hysterectomies to detect and address possible recurrences.

## 1. Introduction

Adenomyosis is characterized by the invasion of endometrial glands into the myometrium, accompanied by muscle hyperplasia.^[1]^ The prevalence of adenomyosis ranges widely, from 5% to 70%, with histological examination being the previous gold standard for diagnosis.^[2]^ Adenomyosis presents various menstrual symptoms, such as heavy menstrual bleeding, dysmenorrhea, pelvic pain, and dyspareunia.^[3]^ The treatment options for adenomyosis encompass both conservative and surgical approaches. Conservative management involves using nonsteroidal anti-inflammatory drugs and hormonal contraceptives for symptomatic patients who prefer nonsurgical interventions.

Endometrial ablation, endometrial resection, electrocoagulation, myometrial excision, and adenomyoma excision are commonly employed to alleviate symptoms associated with adenomyosis.^[2]^ Hysterectomy, either total or supracervical, represents the definitive treatment for adenomyosis. Total or supracervical hysterectomy is generally effective in reducing pain and bleeding symptoms.^[4]^ However, a supracervical hysterectomy has potential drawbacks, including postoperative cyclic bleeding and an increased risk of cervical stump carcinoma compared to a total hysterectomy.

A previous study reported a case of late recurrence of an adenomyoma in the retroperitoneal space 2 years after laparoscopic hysterectomy employing power morcellation.^[5]^ Morcellation has been associated with disseminating benign or malignant tumors within the abdominal cavity.^[6]^ To mitigate this risk, we previously performed manual in-bag morcellation.^[7]^

Given the rarity of recurrent cervical adenomyoma recurrence following a supracervical hysterectomy, we report the case of a woman experiencing recurrent adenomyoma 7 years after a supracervical hysterectomy.

## 2. Case presentation

The written informant consent was obtained from the patient herself.

**Figure 1.**
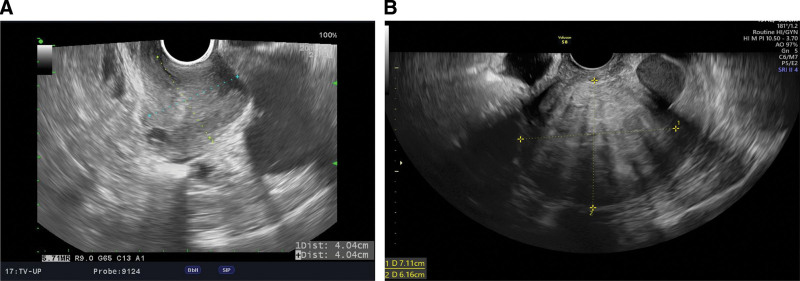
Pelvic ultrasound images of the cervix after supracervical hysterectomy. (A) Remnant cervix after supracervical hysterectomy. (B) Cervical adenomyoma developed 7 years after the first surgery.

A 47-year-old woman presented to our clinic complaining of increased vaginal bleeding last year. The patient had a history of laparoscopic supracervical hysterectomy performed 7 years ago to address symptoms of menorrhagia and dysmenorrhea attributed to uterine myoma and adenomyosis. One month after the hysterectomy, a pelvic ultrasound revealed a cervical stump measuring approximately 4.0 × 4.0 cm (Fig. 1A). Subsequent follow-up pelvic ultrasound revealed progressive growth of the cervical mass, reaching a size of approximately 5.6 × 5.6 cm. Two years ago, a recurrent myoma measuring about 4.6 × 3.8 cm was identified and continued to enlarge. The patient recently reported episodes of intermittent vaginal bleeding. Over 7 years, the cervical mass gradually increased from 4 to 7 cm. Pelvic ultrasonography confirmed the presence of cervical adenomyosis measuring 7 × 6 cm (Fig. 1B).

The patient had a medical history of thyroid cancer 5 years ago, managed by undergoing total thyroidectomy followed by thyroxine treatment. She had also undergone surgery 8 years ago for a benign tumor of the left breast. Her hypertension was well-controlled through regular medical controls. The patient was nulliparous (gravida 0, para 0, abortions 0). Her mother had a history of breast, lung, and thyroid cancer, while her father had hypertension. Additionally, 2 of her sisters had breast cancer.

On physical examination, the patient presented with a soft, non-tender abdomen without any palpable mass. Pelvic examination revealed a pinpoint os and an enlarged cervix.

Her hemoglobin was 13.8 g/dL (within the normal range of 12–16), and her hematocrit level was 39.9% (within the normal range of 36–46). The levels of aspartate aminotransferase and alanine transaminase were 19 U/L (normal < 39) and 35 U/L (normal < 52), respectively. Her blood urea nitrogen and creatinine levels were 15 (normal value < 25) and 0.75 mg/dL (normal value < 1.2), respectively.

After a supracervical hysterectomy, pelvic ultrasonography revealed a remaining cervix measuring 4 × 4 cm (Fig. 1A). Seven years later, before admission, pelvic ultrasonography revealed cervical adenomyosis (7 × 6 cm) with a heterogeneous appearance (Fig. 1B)—recurrent adenomyoma after supracervical hysterectomy was then diagnosed.

**Figure 2.**
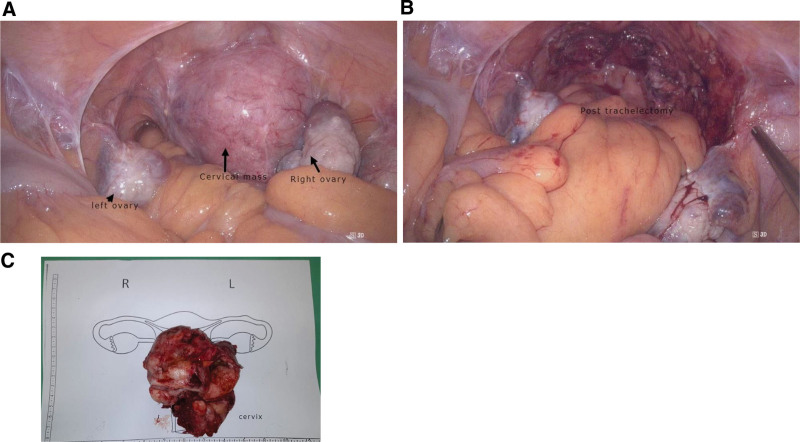
Laparoscopic-assisted vaginal trachelectomy for cervical adenomyoma. (A) Laparoscopic view of the pelvic cavity before trachelectomy. (B) Laparoscopic view of the pelvic cavity after trachelectomy. (C) Specimen of cervical adenomyoma.

**Figure 3.**
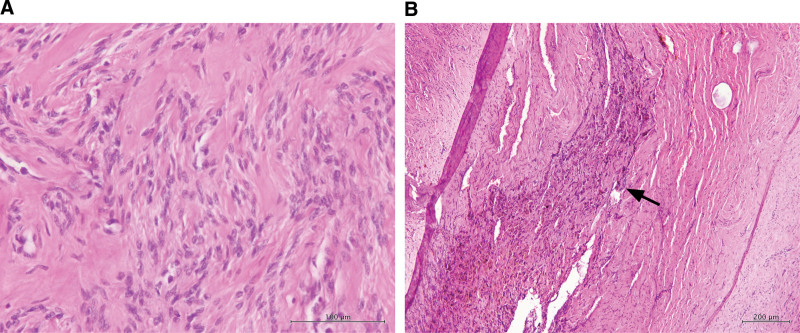
Histopathological examination of the adenomyoma. (A) Bland spindle cells exhibit a fascicular pattern with a cigar or oval-shaped nuclei. Scale bar = 100 μm. (B) Adenomyosis is characterized by hemorrhage in the myometrium (arrow). Scale bar = 200 μm.

The patient then underwent a laparoscopic trachelectomy. Intraoperatively, an enlarged cervix measuring approximately 7 × 6 cm, exhibiting signs of adenomyosis and uterine myoma, was observed (Fig. 2A). The bilateral adnexa appeared normal. Laparoscopy-assisted vaginal trachelectomy was performed (Fig. 2B) with an estimated blood loss of 350 mL. A gross examination of the specimen revealed hypertrophic muscle tissue and hemorrhagic foci (Fig. 2C). Histopathological examination confirmed the presence of adenomyoma (Fig. 3).

The patient continued to have a favorable condition, with no signs of recurrence during the 8-month postoperative follow-up. There were no reports of vaginal bleeding or compression symptoms during this period. The healing progress of the vaginal cuff appeared to be satisfactory. Ultrasonography results were consistently negative. However, the patient did experience urinary frequency after the surgery. To address this, Solifenacin (Astellas Pharma Inc., Kyoto, Japan) was prescribed at a daily dosage of 1 tablet for 3 months, which successfully resolved the issue.

## 3. Discussion

We report an uncommon recurrent adenomyoma in the remaining cervix, which manifested 7 years after a previous supracervical hysterectomy. The patient experienced intermittent vaginal bleeding in recent years, leading to a decision for a trachelectomy. Postoperatively, the patient exhibited a favorable outcome, with no recurrence observed during the 5-month follow-up period.

Secondary resection of the cervical stump after supracervical hysterectomy has been analyzed in a previous retrospective study.^[8]^ The study identified 3 primary indications for a second surgery: symptomatic pelvic organ prolapses (31.4%), spotting (19.0%), and dysplasia or suspicious Pap smear results (18.2%). Only a small proportion of cases were attributed to leiomyoma (3.7%) and endometriosis (5.1%). The median time between the initial supracervical hysterectomy and the secondary surgery was 40 months, with a wide range spanning from 0 to 647 months.

Cervical adenomyoma recurrence may have several causes, including power morcellation and remnant cervical adenomyosis or myoma. Several studies have reported myoma recurrence in the pelvic cavity after power morcellation.^[5,9,10]^ To mitigate this risk, we adopted the practice of manual in-bag morcellation in our previous procedures.^[7]^ Our case is likely caused by remnant cervical adenomyosis.

Table 1 summarizes the current literature review regarding the patient’s characteristics of recurrent adenomyoma/myoma after supracervical hysterectomy.^[5,8,11–15]^ The age range of 39 to 55 years is common for the recurrence of cervical adenomyoma. The symptoms of recurrent cervical stump tumors include pelvic pain, abdominal discomfort, abdominal distention, vaginal bleeding, and constipation. The timing of recurrence varies from 2 to 8 years after the initial procedure. Managing recurrent cervical stump tumors encompasses laparotomy, laparoscopy, and robotic surgery. Pathological findings from reported cases demonstrated myoma in ten cases, adenomyosis in 8 cases, and adenomyoma in 1 case.

**Table 1:** The summary of previous literature regarding cervical tumor recurrence after previous supracervical hysterectomy.

Author, years	Age (years)	Recurrence years after last op	Symptoms	Tumor size (cm)	Treatment	Pathology
Chu et al (2012)^[11]^	50	7	Pelvic pain, vaginal bleeding	15	Laparotomy	Myoma
	55	8	Pelvic pain, vaginal bleeding	11	Laparoscopy	Myoma
Zaki et al (2021)^[5]^	45	2	Constipation, pelvic pain	4.5	Laparoscopy	Adenomyosis
Hilger and Magrina (2006)^[12]^	44	5	Pelvic pain	3	Robotic assisted resection of the pelvic mass and trachelectomy	Myometrium and adenomyosis
Giles (1923)^[13]^	39	7	Abdominal swelling, abdominal discomfort	A hard tumor was filling with true pelvis and arising up to abdomen	Laparotomy	Myoma
Mathew and Abraham (2018)^[14]^	68	Unknown	Lower abdominal pain, abdominal distention	No recorded	Exicion of tumor	Myoma
Krishnamoorthy (2018)^[15]^	40	6	Abdominal distention	30wks pregnant uterus	Laparotomy	Myoma
Neis et al (2021)^[8]^	Average 52.3	40 months	–	–	Vagina in 75.2% cases, laparoscopic in 20.4% of cases, and abdominal in 4.4% of cases.	Myoma 5/137 (3.7%) endometriosis 7/137 (5.1%)
Current case (2023)	47	7	Vaginal bleeding	7	Laparoscopy	Adenomyoma

Ultrasonography is a commonly used diagnostic tool to detect adenomyomas. Adenomyomas are benign uterine tumors of glandular and muscular tissues.^[16]^ Ultrasound imaging can provide valuable information regarding the size, location, and characteristics of adenomyomas. Transvaginal ultrasonography is often the preferred modality for evaluating adenomyomas as it provides detailed imaging of the uterus.^[16]^ During this procedure, a vaginal probe is used to obtain clearer images of the pelvic organs than to abdominal ultrasound. Adenomyomas typically appear as well-defined round or oval masses within the uterine wall. They may exhibit a heterogeneous appearance due to the combination of glandular and muscular tissues.^[17]^

Magnetic resonance imaging is highly sensitive in distinguishing between different types of uterine pathologies, such as myomas, adenomyosis, and malignant changes, particularly in cases where a cervical mass is detected after supracervical hysterectomy. The diagnostic performance of magnetic resonance imaging, as measured by the area under the curve, is 0.91 for detecting myomas, 0.73 for detecting adenomyosis, and 0.80 for detecting leiomyosarcomas.^[18]^

Supracervical hysterectomy is a commonly performed gynecological procedure, offering benefits such as shorter operation times and lower complication rates.^[19]^ However, cyclic bleeding may be a disadvantage associated with supracervical hysterectomies. Clinicians should be aware of the possibility of postoperative recurrence in the cervical stump, particularly in symptomatic patients with a history of adenomyosis and myoma. It is also important to consider the potential for malignancy when evaluating pelvic masses. A study by Neis et al reported a 2.9% incidence of cancer development occurring more than 5 years after supracervical hysterectomy.^[8]^ Our study reminds us that patients who received a supracervical hysterectomy should be followed up with an ultrasound and pap smear to rule out the possibility of recurrence and malignant changes in the cervical stump.

There were several limitations in this case report. This report was based on a single patient’s experience. As such, the findings and conclusions may only apply to some patients undergoing a similar procedure. This case report lacked comparative data with other patients who may have undergone different treatments or experienced similar conditions. Comparative data would enable a better assessment of the treatment’s effectiveness. While this case report provided valuable insights, the findings may need to be more generalizable to a broader population of patients due to the unique nature of the case. The report highlighted the need for more extensive research and case series to establish standardized guidelines for managing cervical adenomyoma following supracervical hysterectomies. Addressing these limitations would provide a more comprehensive understanding of cervical adenomyoma and its management.

## 4. Conclusions

Recurrent cervical adenomyosis and leiomyoma represent uncommon yet potential complications following supracervical hysterectomy. Diagnosing recurrent cervical adenomyosis and myoma can pose challenges and require a vigilant approach. The management of cervical adenomyosis should be tailored based on symptom severity and the extent of the disease. In some cases, combining hormonal therapy with surgical interventions may be necessary to alleviate symptoms and prevent recurrence effectively. Clinicians should maintain a high level of suspicion for cervical adenomyosis/leiomyomas in patients with persistent menstrual pain and bleed after a supracervical hysterectomy. Early recognition and appropriate management can significantly improve patient outcomes in these cases.

## Acknowledgments

We thank Dr Chiu-Hsuan Cheng for her assistance with the pathological pictures.

## Author contributions

**Conceptualization:** Dah-Ching Ding.

**Data curation:** Chin-Tzu Tien, Dah-Ching Ding.

**Funding acquisition:** Dah-Ching Ding.

**Formal analysis:** Chin-Tzu Tien.

**Investigation:** Dah-Ching Ding.

**Methodology:** Chin-Tzu Tien, Dah-Ching Ding.

**Project administration:** Dah-Ching Ding.

**Resources:** Dah-Ching Ding.

**Supervision:** Dah-Ching Ding.

**Validation:** Dah-Ching Ding.

**Visualization:** Dah-Ching Ding.

**Writing – original draft:** Chin-Tzu Tien, Dah-Ching Ding.

**Writing – review & editing:** Dah-Ching Ding.
